# Single-cell RNA sequencing reveals that targeting HSP90 suppresses PDAC progression by restraining mitochondrial bioenergetics

**DOI:** 10.1038/s41389-021-00311-4

**Published:** 2021-03-03

**Authors:** Li-Peng Hu, Kai-Xia Zhou, Yan-Miao Huo, De-Jun Liu, Qing Li, Min-Wei Yang, Pei-Qi Huang, Chun-Jie Xu, Guang-Ang Tian, Lin-Li Yao, Xue-Li Zhang, Ya-Hui Wang, Jun Li, Zhi-Gang Zhang, Shu-Heng Jiang, Xin Xing, Xu Wang, Wei-Ting Qin, Qin Yang

**Affiliations:** 1 State Key Laboratory of Oncogenes and Related Genes, Shanghai Cancer Institute, Ren Ji Hospital, School of Medicine, Shanghai Jiao Tong University, Shanghai, P.R. China; 2 Department of Biliary-Pancreatic Surgery, Ren Ji Hospital, School of Medicine, Shanghai Jiao Tong University, Shanghai, P.R. China; 3 Shanghai Fengxian District Central Hospital, Shanghai, P.R. China; 4 Department of Radiation Oncology, Institute of Oncology, Affiliated Hospital of Jiangsu University, Zhenjiang, P.R. China

**Keywords:** Pancreatic cancer, Cancer metabolism

## Abstract

Pancreatic ductal adenocarcinoma (PDAC) is one of the deadliest cancers, which lacks effective treatment strategies. There is an urgent need for the development of new strategies for PDAC therapy. The genetic and phenotypic heterogeneity of PDAC cancer cell populations poses further challenges in the clinical management of PDAC. In this study, we performed single-cell RNA sequencing to characterize PDAC tumors from KPC mice. Functional studies and clinical analysis showed that PDAC cluster 2 cells with highly Hsp90 expression is much more aggressive than the other clusters. Genetic and pharmacologic inhibition of Hsp90 impaired tumor cell growth both in vitro and in vivo. Further mechanistic study revealed that HSP90 inhibition disrupted the interaction between HSP90 and OPA1, leading to a reduction in mitochondrial cristae amount and mitochondrial energy production. Collectively, our study reveals that HSP90 might be a potential therapeutic target for PDAC.

## Introduction

Pancreatic ductal adenocarcinoma (PDAC) is one of the most aggressive cancers and is projected to be the second highest contributor to cancer-related deaths by 2030^1^. The lack of both early diagnosis and appropriate targeted therapies leads to an extremely low survival rate in PDAC patients^2,3^. Despite decades of extensive work toward improving diagnostic techniques, surgical procedures, and chemotherapy, the prognosis of PDAC patients is still poor, and the average 5-year survival rate is less than 8%^4^, indicating that the development of new targeted therapies or effective interventions is urgently needed.

PDAC is a highly heterogeneous disease with numerous genetic alterations^5,6^. Gene mutations, epigenetic changes, and copy number alterations facilitate clonal selection and finally contribute to the malignant phenotype of cancer cells^7^. Owing to current technology development, the global gene expression profiles of single cells from a bulk tumor could be defined, facilitating dissection of heterogeneity in cell populations that was previously hidden^8^, which might provide potential prognostic biomarkers and guide better clinical decisions for personalized treatment.

Heat shock protein 90 (HSP90), encoded by *HSP90AA1*, plays a crucial role in both physiological and stress conditions^9^. Previous studies have reported that HSP90 is widely involved in many human diseases, including cancer, neurodegenerative diseases, and cystic fibrosis^10^. In prostate cancer, HSP90 enhances castrate resistance by interacting with the androgen receptor^11,12^. In breast cancer, HSP90 binding to mutant p53 leads to an accumulation of dysfunctional p53 in cancer cells^13^. HSP90 can also directly interact with the DNA methyltransferase DNMT1 to regulate the viability of leukemia cells^14^. Although HSP90 has been studied extensively in cancer, its role in mitochondrial oxidative phosphorylation remains unknown.

In this study, by utilizing single-cell RNA sequencing, we aimed to determine the intratumoral heterogeneity of murine PDAC and screen potential targets for PDAC therapy. Our data showed that HSP90 conferred PDAC cell growth advantages. Targeting HSP90 with BIIB021 disrupts the interaction between HSP90 and OPA1, resulting in cristae shrinkage and reduced mitochondrial energy production. Together, our data suggest that HSP90 inhibition prevents PDAC progression by restraining mitochondrial bioenergetics.

## Results

### Single-cell expression profiling and cell typing in pancreatic tumors from KPC mice

**Fig. 1:**
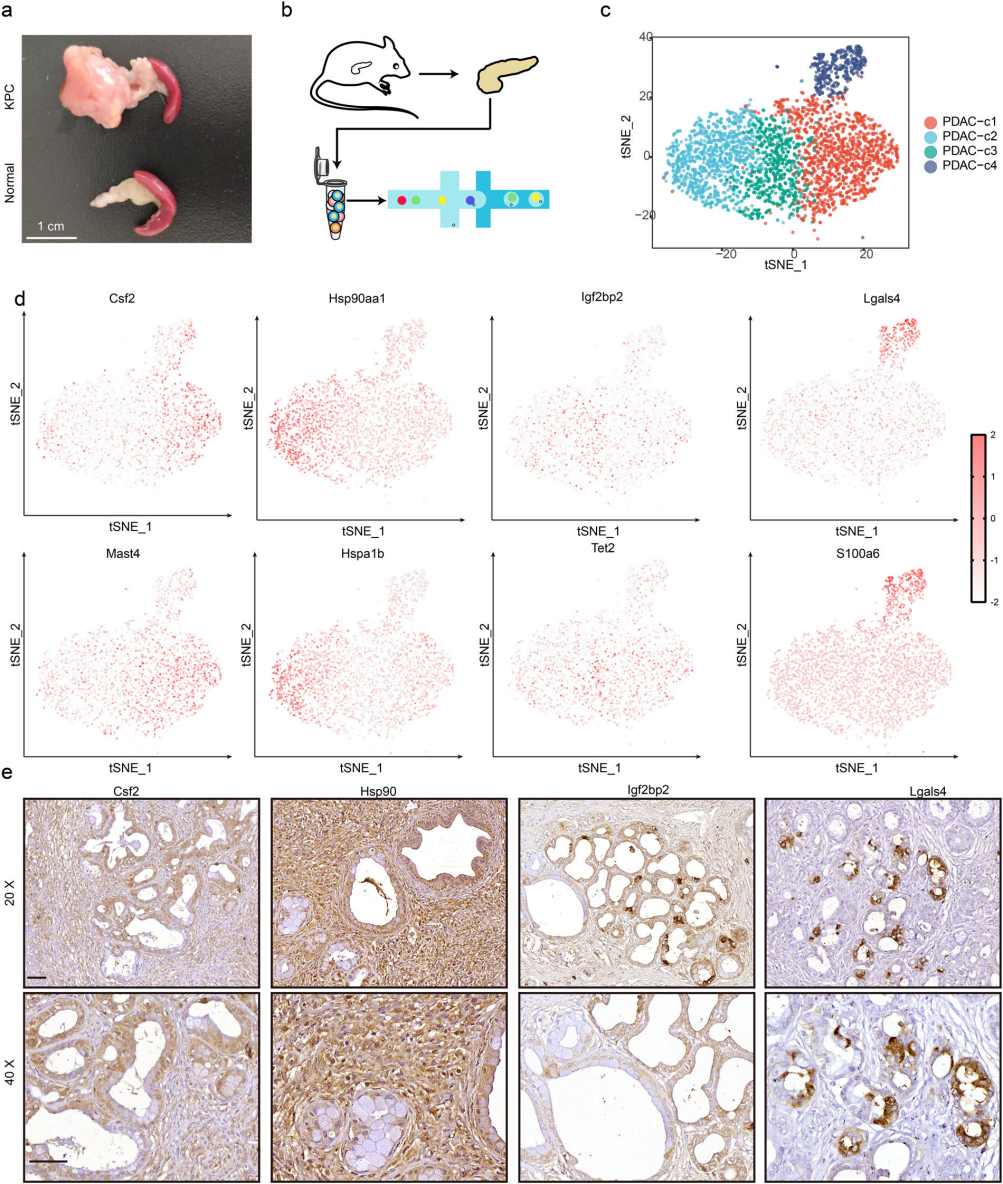
Single-cell RNA sequencing analysis of tumors derived from KPC mice. **a** The comparison of pancreas size from KPC mice and the littermate control. **b** Procedure of KPC tumors for single-cell RNA sequencing. **c** The t-distributed stochastic neighbor embedding (t-SNE) plot of KPC cells. **d** Expression levels of representative markers for the four types of KPC tumor cells. **e** Representative marker IHC staining of four PDAC clusters. Scale bar, 50 μm.

To explore cellular diversity in murine PDAC, we generated single-cell RNA-seq profiles from the solid tumors of murine PDAC tumor samples derived from *Kras*^*+/LSL-G12D*^; *Trp53*^*+/LSL-R172H*^; *Pdx1-Cre* (KPC) mice, which well recapitulate the progression of human PDAC and are widely used for PDAC drug evaluation (Fig. 1a, b). After initial quality control, we acquired single-cell transcriptomes in a total of 4084 cells from the solid tumor. Cumulatively, cells with low expression of genes (<300 genes) and a high percentage of mitochondrial genes expressed (>10%) were digitally filtered out, resulting in 3763 single cells used for the subsequent analysis. To explore the cellular composition of tumors, two-dimensional t-distributed stochastic neighbor embedding (t-SNE) was applied to variably expressed genes across all cells, and seven main clusters were identified, including acinar cells, endothelial cells, fibroblast cells, and PDAC tumor cells, and these clusters could be further divided into four clusters (Fig. 1c and Supplementary Fig. 1a, b). We found that PDAC cluster 1 and PDAC cluster 2 were the dominant subgroups, accounting for 35% and 26.84% of the total tumor cells, respectively. However, the PDAC cluster 3 and PDAC cluster 4 populations were relatively limited, only comprising 17.59% and 10.26% of all tumor cells, respectively (Supplementary Fig. 1b). We also compared the clusters identified by scRNA sequence with clusters that achieved with deconvolution algorithms a la Moffitt. The results showed that all the scRNA clusters contain both basal and classical. The C1 and C4 were presented more classical type. The proportion of basal in C3 cluster is higher than classical type. As for C2, the proportion of basal and classical is very close to each other (Supplementary Fig. 1c). By comparing the gene expression patterns, specific genes were identified that could be used to distinguish these subgroups. *Csf2* and *Mast4* were mainly expressed in PDAC cluster 1, and *Hsp90aa1* and *Hspa1b* were dominantly expressed in PDAC cluster 2. Igf2bp2 and *Tet2* were biomarkers of PDAC cluster 3. *Lgals4* and *S100a6* were differentially expressed in cluster 4 (Fig. 1d and Supplementary Fig. 1d, e). Next, we further validated these subsets of cells in vivo by immunohistochemistry staining (Fig. 1e). The results showed that Csf2 mainly expressed in pancreatic intraepithelial neoplasia (PanIN) and PDAC cells. Comparatively, Hsp90 dominative expressed on PDAC cells, barley expressed in PanIN cells. In addition, Igf2bp2 expressed in both low- and high-grade PanIN cells, Lgals4 mainly expressed in low-grade PanIN cells.

### PDAC cluster 2 is the most aggressive type of the four PDAC clusters

**Fig. 2:**
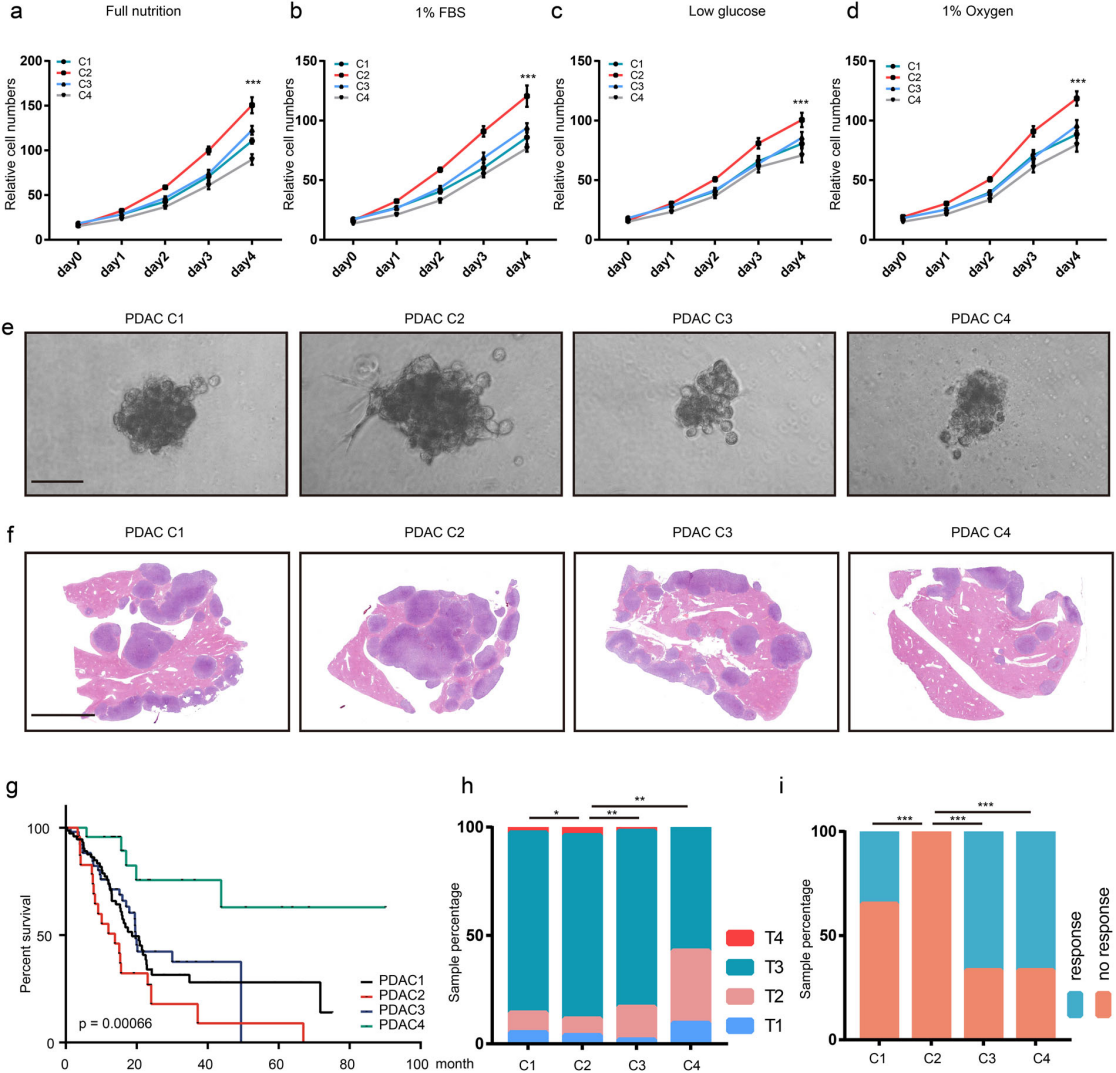
PDAC cluster 2 is the most aggressive type of four PDAC clusters. **a**–**d** Cell proliferation assay of four cluster cells in full nutrition (**a**), 1%FBS (**b**), low glucose (0.46 mg/L) (**c**), or 1% oxygen (**d**) condition. **e** 3D on-top growth of four cluster cells. **f** Liver metastasis of four cluster cells. **g** Kaplan–Meier survival plot of the four clusters defined in TCGA cohort. **h** Tumor stage percentage of the four clusters defined in TCGA cohort. **i** Chemotherapy response of the four clusters defined in TCGA cohort.

To gain further insight into the differences among the four PDAC clusters, four cell lines with represented gene expression pattern (Supplementary Fig. 2a) derived from KPC tumor were used to compare their tumorgenicity ability, named as C1, C2, C3, and C4. Cell proliferation assay results showed that C2 cell lines presented growth advantages compared with the other cell lines in both full nutrition condition and poor nutrition condition (Fig. 2a–d). Next, 3D on-top growth assay was applied to mimic the cancer cell spatial growth condition (Fig. 2e). Consistently, the cell spheres of C2 were much bigger than that derived from other cluster cells. Furthermore, the metastatic propensity of four cluster cells was evaluated by taking use of intrasplenical injection derived PDAC liver metastasis model. The results showed that the percentage of invaded liver area by C2 cell was much higher than the other cluster cells (Fig. 2f and Supplementary Fig. 2b). In addition, gene set variation analysis (GSVA) was performed to score and divide PDAC cohort of the Cancer Genome Atlas (TCGA) samples into four groups depending on the gene expression pattern of the four mouse PDAC clusters identified from the mouse single-cell sequencing data (Supplementary Table 1). Next, we compared the survival rate between different groups of PDAC patients via the Kaplan–Meier method and log-rank tests and found that PDAC cluster 2 patients had the shortest survival time compared to the other clusters (Fig. 2g). Also, we noticed that the patients in cluster 2 of TCGA cohort exhibited higher tumor stage and poorer chemotherapy response (Fig. 2h, i). Taken together, those data indicated that PDAC cluster 2 was most aggressive than the other three clusters.

### HSP90 inhibition impairs tumor cell growth by restraining mitochondrial bioenergetics

**Fig. 3:**
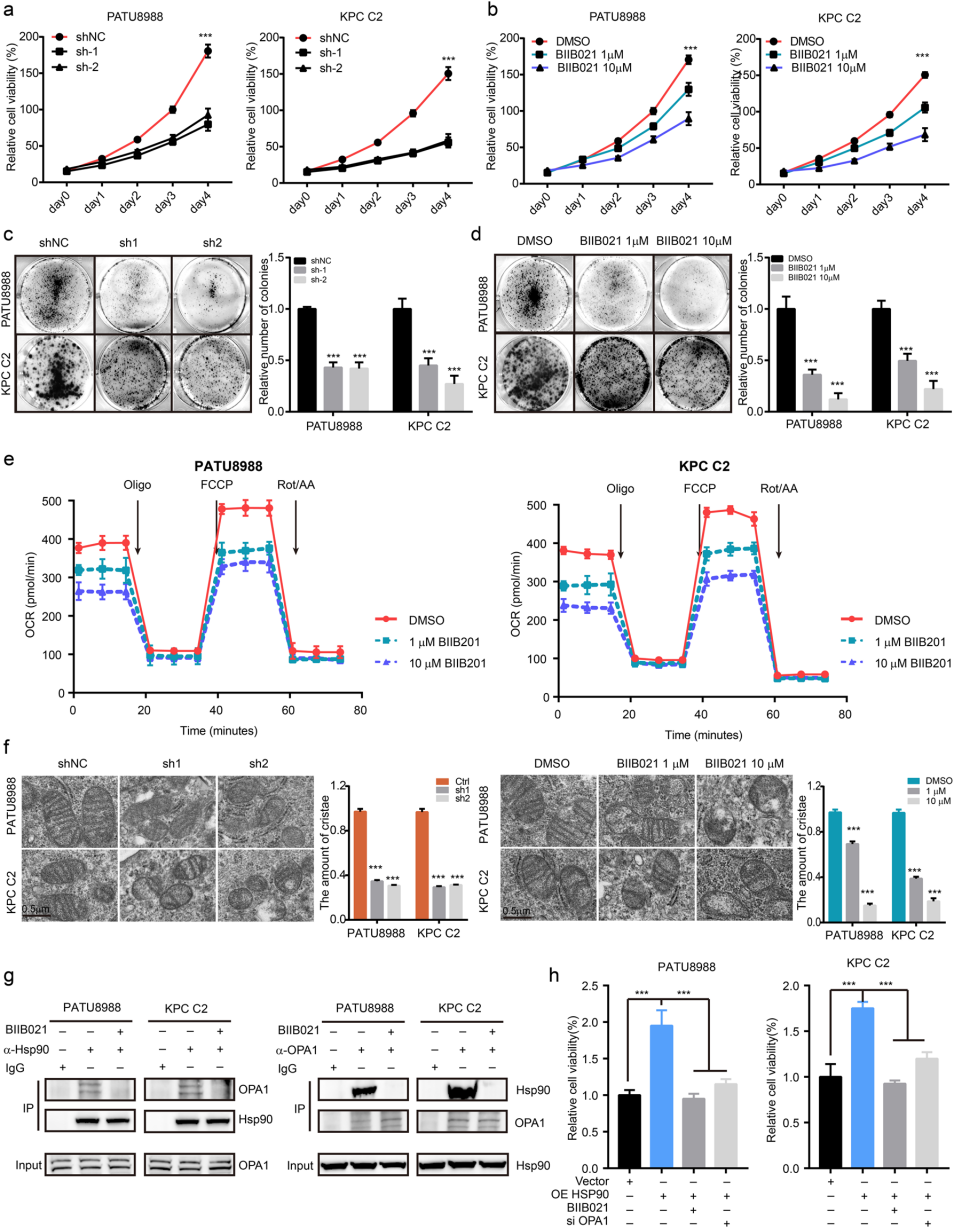
HSP90 inhibition suppressed mitochondrial energy production in PDAC. **a** Relative cell viability of PATU8988 cells stably expressing shNC, shHSP90AA1-1 (named sh1), and shHSP90AA1-2 (named sh2) and relative cell viability of KPC C2 cells stably expressing shNC, sh*Hsp90aa1*-1 (named sh1), and sh*Hsp90aa1*-2 (named sh2). Statistical results vs. the shNC group. **b** Relative cell viability of PATU8988 and KPC C2 cells treated with DMSO, 1 μM BIIB021 or 10 μM BIIB021. Statistical results vs. the DMSO group. **c** Colony formation assay of PATU8988 and KPC C2 cells stably expressing shNC, sh1, and sh2. **d** Colony formation assay of PATU8988 and KPC C2 cells treated with DMSO or different doses of BIIB021. **e** OCR measurement in human and mouse PDAC cells treated with DMSO, 1 μM BIIB021 or 10 μM BIIB021. **f** Morphometric analysis of mitochondria with transmission electron micrographs upon HSP90 inhibition. **g** Coimmunoprecipitation of HSP90 and OPA1 in PDAC cell lines treated with or without BIIB021 for 24 h. **h** Cell proliferation assay of PDAC cells with HSP90 overexpression plus BIIB021 or OPA1 siRNA.

Thus, we aimed to reveal the underlying reasons why cluster 2 PDAC was much more aggressive. The differential gene expression analyses showed that *Hspa1b* and *Hsp90aa1* were the most significantly expressed genes in cluster 2 compared to other clusters (Supplementary Fig. 3a). Considering that HSPA1B was not clinically relevant to prognosis, we further focused on the roles of HSP90AA1 in PDAC. By analyzing GEO data sets, we found that *HSP90AA1* mRNA expression levels were greatly upregulated in PDAC compared to adjacent tissues (Supplementary Fig. 3b). Next, IHC results from KPC-derived tumors showed that HSP90 protein expression was elevated in PanINs and PDAC tissues in a stepwise manner (Supplementary Fig. 3c). Furthermore, a clinical PDAC tissue array (named as Renji cohort) showed that HSP90 expression was significantly upregulated in PDAC tissues compared to adjacent tissues (Supplementary Fig. 3d), and Kaplan–Meier analysis further revealed that high expression of HSP90 in cancer tissues was associated with a poor prognosis in both the Renji and TCGA cohorts (Supplementary Fig. 3e, f). To investigate the function of HSP90AA1 in PDAC cluster 2 cancer cells, we silenced HSP90AA1 in human PDAC cell line PATU8988 and mouse PDAC cell line KPC C2 by stably expressing short hairpin RNA (shRNA) (Supplementary Fig. 3g). HSP90AA1 knockdown significantly inhibited cell proliferation (Fig. 3a). Next, we treated those cells with different concentrations of BIIB021, a specific inhibitor of HSP90 (1–10 μM). BIIB021 markedly reduced the viability and induced the apoptosis of PATU8988 and KPC C2 cells in a dose-dependent manner (Fig. 3b and Supplementary Fig. 4a, b). In line with this, the colony formation assay results indicated that HSP90 inhibition greatly suppressed PATU8988 and KPC C2 cancer cell clonogenicity ability (Fig. 3c, d).

Next, we aimed to elucidate the underlying mechanisms by which HSP90 inhibition affected PDAC cancer cell proliferation. PDAC patients of TCGA cohort were divided into HSP90-low group and HSP90-high group depended on the mRNA level of HSP90AA1, following gene set enrichment analysis. The results revealed that the gene sets related to mitochondrial bioenergetics, including oxidative phosphorylation, citrate cycle TCA cycle, and Pyruvate metabolism, were enriched in samples with high HSP90 expression (Supplementary Fig. 4c). Thus, we next explored whether mitochondria were involved in the impaired cell viability induced by HSP90 inhibition. ATP production assay results showed that genetic and pharmacologic inhibition of HSP90 markedly reduced ATP production in PDAC cells (Supplementary Fig. 4d), indicating that mitochondria function was debilitated by HSP90 inhibitor. However, mitochondrial DNA assessment indicated that the amounts of mitochondria exhibited only a slight decrease upon HSP90 inhibition (Supplementary Fig. 4e). Thus, we further detected mitochondrial oxidative phosphorylation in PDAC cells upon HSP90 inhibition by measuring the oxygen consumption rate (OCR). The results showed that HSP90 silencing with shRNA also significantly reduced the OCR in both human and mouse PDAC cells (Supplementary Fig. 4f). In line with this, BIIB021 treatment greatly inhibited the OCR of PDAC cells in a dose-dependent manner (Fig. 3e). In addition, morphometric analysis of mitochondria by electron micrographs found that Hsp90 inhibition markedly reduced mitochondrial cristae levels (Fig. 3f). In addition, we observed that the mitochondrial membrane potential significantly decreased upon HSP90 silencing or blockade with BIIB021 (Supplementary Fig. 5a). Considering the dominative roles of OPA1 in mitochondrial cristae remodeling^15^, HSP90 inhibition may disturb the function of OPA1 to regulate mitochondrial cristae remodeling in PDAC cells. Endogenous immunoprecipitation assays showed that HSP90 interacted with OPA1 in both human and mouse PDAC cancer cells and this interaction was disrupted with BIIB021 administration (Fig. 3g). To further confirm that OPA1 was involved in the HSP90-mediated PDAC growth advantage, OPA1 siRNA was applied to HSP90-overexpressing PDAC cells (Supplementary Fig. 5b). The data showed that OPA1 silencing counteracted the enhanced mitochondrial oxidative phosphorylation and growth promotive effects mediated by HSP90 overexpression (Fig. 3h and Supplementary Fig. 5c). Taken together, HSP90 inhibition impaired the interaction between HSP90 and OPA1, resulting in cristae remodeling and energy production suppression in mitochondria.

### HSP90 inhibition suppresses the progression of PDAC in vivo

**Fig. 4:**
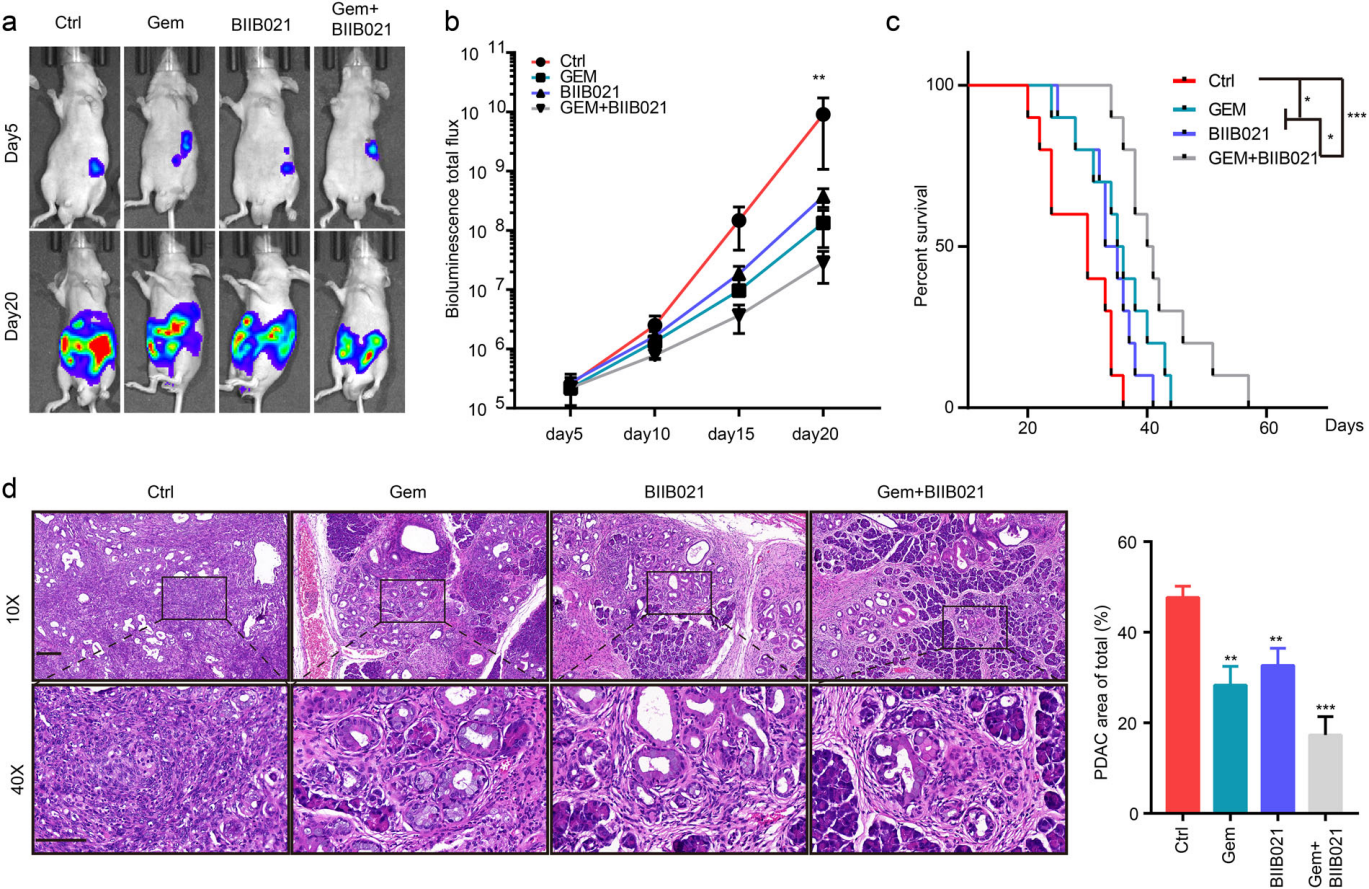
HSP90 inhibition suppress the progression of PDAC in vivo. **a** Representative bioluminescence photograph of mice orthotopically implanted with KPC C2 Luc cells treated with 0.9% NaCl (named ctrl), gemcitabine (50 mg/kg), BIIB021 (10 mg/kg), or gemcitabine (50 mg/kg) plus BIIB021 (10 mg/kg). **b** Statistics for the total bioluminescence of the orthotopic PDAC mouse model (*n* = 5). **c** Kaplan–Meier survival plot of orthotopic PDAC mice (*n* = 10 per group). **d** H&E staining of KPC mice treated with 0.9% NaCl (named ctrl), gemcitabine (50 mg/kg), BIIB021 (10 mg/kg), or gemcitabine (50 mg/kg) plus BIIB021 (10 mg/kg). Scale bar, 50 μm. The statistical results of the PDAC area are shown in the right panel (*n* = 5).

To further evaluate the role of HSP90 in tumor growth and maintenance in vivo, an orthotopic PDAC mouse model was generated by injecting Luc-expressing KPC cells into the pancreas of nude mice. Total luminescence flux derived from bioluminescence imaging was used to evaluate orthotopic tumor growth. Subsequent BIIB021 administration delayed the growth rate of tumor growth compared to control mice (Fig. 4a). The combination of BIIB021 and gemcitabine greatly reduced the tumor burden and led to a significant extension of median survival from 21.86 to 44.86 days (Fig. 4b, c). In addition, the anti-tumor effects of BIIB021 were also evaluated in KPC mice. BIIB021 alleviated the development of tumors in KPC mice and exhibited additive effects with gemcitabine (Fig. 4d). Collectively, our results demonstrated that targeting HSP90 with BIIB021 effectively prevented PDAC progression.

## Discussion

During cancer development, the heterogeneity of cancer cells gradually expands. Accumulating studies have reported that therapeutic responses are largely determined by the evolution of subpopulations and changes in cellular phenotypes^16,17^. Thus, unraveling tumoral heterogeneity is necessary, laying the foundation for the development of more effective therapies. In this study, we explored the cellular heterogeneity of cancer cells in KPC mouse model. Our data showed that murine PDAC tumor cells could be subdivided into four groups according to single-cell RNA-seq. Of note, four pancreatic ductal tumor cell types exhibited highly different transcriptomics. Previous human PDAC specimen scRNA-seq analysis reported two ductal cell types: normal ductal cell and tumor ductal cell^18^. In addition, Elyada et al. resected KPC tumors to perform single-cell analysis^19^. However, they mainly focused on the heterogeneity of cancer-associated fibroblasts and showed many immune cells in KPC tumors. Two reasons may account for this difference: One reason is that the time points of KPC tumor cell resection are different. The tumors in our study were resected at a relatively late stage, and the mice exhibited obvious cachexia with 25% weight loss compared to their littermates. Relatively fewer immune cells infiltrated into the interior of large tumors. The other reason is that the time of enzymatic digestion is different. To fully dissociate cancer cells from the tumor, we performed three digestion cycles at 30 min per cycle, and the immune cells were largely lost during this process.

Previous studies have reported that HSP90 is involved in pancreatic cancer chemotherapy and radiotherapy resistance^20^ and JAK-STAT3 signaling regulation^21^. In our study, by using our data combined with TCGA PDAC patient transcriptomic and clinical data, we found that the high-*HSP90AA1* expressing PDAC cluster 2-related genes exhibited the shortest overall survival time. Further analysis showed that high *HSP90* expression was associated with poor prognosis in both the TCGA cohort and Renji cohort. Consistent with our observation, higher HSP90 expression was also associated with shorter overall survival time in hepatocellular carcinoma, colorectal cancer, gastric cancer, and breast cancer. Owing to its widespread expression in cells and more than 200 client-proteins^22^, HSP90 acts as a crucial regulator of a variety of cellular processes, such as stress responses, cell growth, angiogenesis, and signaling regulation. Recently, the roles of HSP90 in tumorigenesis have gradually attracted researchers’ attention^23^. Cytosol HSP90 can interact with CDC37 to facilitate colorectal cancer progression^24^. Nuclear HSP90 is reported to regulate cell growth-related genes in erythroleukemic cells by stabilizing the transcription factor HCFC1^25^. Mitochondrial HSP90 contributes to vascular remodeling in pulmonary arterial hypertension^26^. In addition, a recent study reported that HSP90 inhibition facilitates the cancer immunotherapy response by upregulating interferon response genes^27^. In this study, our data showed that HSP90 inhibitors BIIB021 significantly reduced the growth and induced the apoptosis of PDAC tumor cells both in vitro and in vivo. Our data showed that the apoptosis was triggered by BIIB021 in PDAC cells; however, we could not complete rule out whether other unclassical cell types, such as ferroptosis, are involved in this process. As for clinical treatment, BIIB021 usage should be personalized, considering that the growth inhibition efficiency of BIIB021 is better in the tumor cells with high HSP90 expression than those cells with low HSP90 expression. Further mechanistic studies revealed that HSP90 can interact with OPA1, the master mitochondria cristae shape regulator, to promote the oxidative phosphorylation and growth of PDAC cells. Considering that both HSP90 and OPA1 are widespread intercellular, more efforts are needed to determine the interaction subcellular location of their interaction, only in the mitochondria or in both the cytosol and mitochondria.

In summary, our work unravels the cellular heterogeneity of KPC tumors. Our data show that high HSP90 expression is associated with poor prognosis. Mechanistic studies revealed that HSP90 interacts with OPA1 to regulate mitochondrial cristae structure and mitochondrial respiration efficiency, indicating that HSP90 could be a potential therapeutic target for PDAC.

## Materials and methods

### Animal study

All animal experiments followed the National Institute of Health guidelines for the Care and Use of Laboratory Animals and were approved by the Institutional Animal Care and Use Committee of East China Normal University. As for orthotopic model, KPC 2-Luc cells (1 × 105) were injected in the pancreas of immunocompromised nude mice. After 1 week, the animals were treated with vehicle (DMSO) 0.9% NaCl, gemcitabine (50 mg/kg), BIIB021 (10 mg/kg), and BIIB021 plus gemcitabine as twice a week i.p. injections for an additional 3 weeks. For liver metastasis modeling, 2 × 105 KPC cells suspended in 20 µl DMEM were implanted into spleen of C57BL/6 mice under 2.5% isoflurane inhalation anesthesia after surgical exposure of the spleen. As for KPC mouse model, the mice were randomly divided into four groups at 10 weeks and treated with vehicle (DMSO) 0.9% NaCl, gemcitabine (50 mg/kg), BIIB021 (10 mg/kg), and BIIB021 plus gemcitabine as twice a week i.p. injections for an additional 6 weeks. For survival analyses, survival was determined by mouse health requiring euthanasia as defined by institutional IACUC guidelines. No mouse tumors exceeded IACUC-defined maximal tumor volumes of ≥1.5 cm^3^. At the end of the experiment, mice in the various groups were sacrificed and their tibias were harvested, fixed in 4% paraformaldehyde for histology assay.

### Single-cell sequencing

Tumors from KPC were minced and digested with tumor dissociation kit (130-096-730, Miltenyi Biotec) for 30 min at 37 °C three cycles. Cells were counted on Countess II automated cell counter (ThermoFisher), and up to 12,000 cells were loaded per lane on 10X Chromium microfluidic chips. Single-cell capture, barcoding, and library preparation were performed using the 10X ChromiumTM version 2 chemistry, and according to the manufacturer’s protocol (#CG00052). cDNA and libraries were checked for quality on Agilent 4200 Tapestation and quantified by KAPA qPCR before sequencing on a single lane of a HiSeq4000 (Illumina).

### PDAC four cluster monoclonal cell lines isolation

Tumors from KPC were minced and digested with tumor dissociation kit (130-096-730, Miltenyi Biotec) for 30 min at 37 °C three cycles. Then, the cell concentration was quantified in this DMEM with a hemocytometer, following diluted the cell solution into the conditioned medium prepared to make a new cell solution at a concentration of 5 cells/ml. And, 100 µl of the 5 cells/ml was transferred into each well of a 96-well plate, minimizing those wells that receive more than one cell.

After the cells have expanded, transfer, and harvested cell to identify the cluster, it belongs to by detecting the marker genes expression level.

### Proliferation assay

Cell viability was measured according to the manufacturer’s instructions of Cell Counting Kit-8 (SB-CCK8S, share-bio, China). Cells with indicated treatment were grown in 96-well plate at 3000 cells per well and cultured for 24, 48, 72, and 96 h. At the indicated time point, the culture medium was removed and 10% (volume/volume) CCK-8 to the culture medium was added to each well. After 1-hour incubation, the optical density was measured at 450 nm using a microplate reader (M1000 PRO, TECAN). The experiments were performed in quintuple manner and repeated twice.

### RNA interference

siRNAs transfection was performed with Lipofectamine^®^ RNAiMAX (Invitrogen, 13778150) following the manufacturer’s instruction. Specific custom OPA1 siRNAs were synthesized in GenePharma (Shanghai, China). As for stable knockdown, shRNAs or shscramble were cloned into pLKO.1 plasmid (Sigma). Lentivirus packaging was performed in 293T cells according to standard protocols. Cells were infected with 1 × 106 recombinant lentivirus-transducing units in the presence of 10 mg/ml polybrene (sigma, H9268). When the confluence up to 40–50%, cells were infected with the indicated supernatant containing viral particles. Puromycin (Gibco, A1113802) was applied to virally infected cells for obtaining stable knockdown or overexpression cell lines. The sequence of HSP90AA1 shRNA is 5’-GCAGCCATTTATATTGCTTAG-3’ and 5’-GCCCTTCTATTTGTCCCACGA-3’. The sequence of Hsp90aa1 shRNA is 5’-GCAACAGCTGAAGGAATTTGA-3’and 5’-GCTGCTGTAACTGAAGAAATG-3’.

### Colony formation assay

In brief, PDAC cells with indicated treatment were seeded in 1000 cells per 2 ml in 6-well plates and the culture medium was replaced every week. All cells were cultured in a humidified incubator containing 5% CO_2_ at 37 °C for the next 3 weeks. At last the colonies were stained with 0.05% (weight/volume) crystal violet in 25% (volume/volume) methanol and counted using Image J software. This experiment was repeated twice.

### 3D on-top growth

3D on-top growth was performed according to standard procedure. A total of 10,00 PDAC cells were seeded into the Matrigel coated plate, following DMEM supplemented with 10% Matrigel was added into the culture plate. Culture medium replaced every 2 days.

### Immunohistochemistry

All antibodies were diluted with PBS (B320KJ, BasalMedia) containing 1% BSA Albumin Fraction V (4240GR250, BioFroxx). Each step was followed by washing with PBS for 10 min each time. For tissue immunohistochemical staining, slides were first deparaffinized in xylene. Next, antigen retrieval was performed by boiling the slides in sodium citrate antigen retrieval solution (YFH5001, YIFAN BIOLOGICAL, China) for 10 min. Then, slides were pretreated with endogenous peroxidase blocking solution (YFH4001, YIFAN BIOLOGICAL, China) for 10 min. After blocking with 10% BSA for 60 min at room temperature, slides were immune-stained with anti-HSP90 antibody (1:100 Proteintech, 13171-1-AP) overnight at 4 °C. The slides were washed thrice of 10 min each time and then with a mixture of HPR-conjugated goat anti-rabbit secondary antibodies (1:300 Jackson ImmunoResearch, 111-035-003) at room temperature for 1 h. After washing thrice, slides were developed in DAB (CST, 8059) for an appropriate time and counterstained with hematoxylin.

### Transmission electron microscopy

The cells fixed in glutaraldehyde were rinsed with sodium cacodylate buffer and then fixed in 1% OsO4 in 0.1 M sodium cacodylate buffer on ice for 2 h before dehydration with acetone. After being embedded in resin, cell pellets were polymerized at 60 °C for 48 h. Ultimately, ultrathin sections (70 nm) were mounted onto copper grids and counterstained with 4% uranyl acetate and lead citrate before observation under a transmission electron microscope (JEM-1230, Japan) operating at 80 kV.

### Western blots

Cells were washed and lysed with RIPA buffer (WB3100, NCM, China) containing protease inhibitors cocktail (B14001, bimake) on ice for 10 min. Then, protein lysate followed centrifugation in 4 °C for 10 min and the supernatant was collected. Protein supernatant were prepared with 5 × SDS loading buffer (P1040, Solarbio) and denatured at 100 °C for 5 min. Appropriate protein of samples were separated by 4–20% Genshare PAGE gel electrophoresis and electroblotted into NC membranes on eBlot™ L1 Protein Transfer System (GenScript). The membranes were incubated in 5% non-fat powdered milk (Cat No. 36101; Yeasen, Shanghai, China) in TBST (TBS with 0.1% Tween20) for 1 h at room temperature, followed by incubation with primary antibodies against specific proteins overnight: β-actin (1:5,000, Yeasen, 30101ES50), HSP90 (1:1000, 13171-1-AP, Proteintech), and OPA1(1:1000, Abcam, ab42364). The primary antibodies were diluted in universal antibody diluent (WB500D, NCM, China). The membranes were washed thrice of 10 min each time and incubated with the HPR-conjugated goat anti-mouse (1:10,000, Jackson ImmunoResearch, 115-035-003) or rabbit secondary antibodies (1:10,000 Jackson ImmunoResearch, 111-035-003) for 1 h at room temperature. Enhanced chemiluminiscence (ECL) was performed using the ECL kit (WB012, share-bio, China), visualized by the Bio-Rad system.

### Immunoprecipitation

Cells with indicated treatments were lysed in IP buffer supplemented with protease inhibitors cocktail (B14001, bimake). Then lysates were incubated with pre-linked anti-OPA1 antibody (Abcam, ab42364), HSP90 (13171-1-AP, Proteintech), or control rabbit IgG (Abcam, ab172730) Dynabeads protein G (Life technologies, 10004D) for 2 h at room temperature, following immunoblot with indicated antibodies.

### Oxygen consumption rate

The assays for OCR of the cultured cells were investigated using a Seahorse XF96 Flux Analyzer (Seahorse Bioscience) according to the manufacturer’s instructions. AsPC-1 and Panc-1 cells were seeded in a XF96-well plate at 1 × 104 per well with the indicated treatment and incubated overnight at 37 °C in a 5% CO_2_ incubator. Prior to measurement, cells were incubated with assay media in a non-CO_2_ incubator at 37 °C for 1 h. Compounds for OCR measurements were added to RPMI 1640 or DMEM assay medium (Sigma-Aldrich) containing oligomycin (1 mM; Sigma-Aldrich), carbonyl cyanide 4-(trifluoromethoxy) phenylhydrazone (FCCP, Sigma-Aldrich, C2920), and antimycin A and rotenone (2 mM; Sigma-Aldrich). The measurement was normalized by total protein quantization. The experiments mentioned above were performed in triplicate and repeated twice.

### Statistical

Data were presented as the mean ± SD or as boxplots and all statistics were conducted using GraphPad Prism 7.0 and Excel. The statistical analysis was performed using one-way ANOVA, two-way ANOVA, or unpaired Student’s *t* test as appropriate for the dataset. The Kaplan–Meier method was used to illustrate the overall survival in patients with PDAC and significance was determined by the log-rank Mantel–Cox test. Functional data are representative of at least triplicates unless otherwise specified. Statistical significance is displayed as *p < 0.05, **p < 0.01, ***p < 0.001, ns: not significant.

### Bioinformatics and data analysis

For scRNA-Seq data sets, 105,781 mean reads per cell and 1390 median genes per cell were obtained. Identification of highly variable genes was performed in Seurat utilizing the MeanVarPlot function using the default settings with the aim to identify the top ∼2000 variable genes. Clustering analysis of single-cell data was performed with Seurat using a graph-based clustering approach. Resolution in the FindClusters function was set to 0.8. Clusters were then visualized using a t-SNE plot. Differential expression analysis was performed in Seurat utilizing the FindAllMarkers function with the default settings except that the “min.pct” and “thresh.use” parameters were utilized to identify broadly expressed (min. pct = 0.25, logfc.threshold = 0.25 = 0.01). The parameter “min.pct” sets a minimum fraction of cells that the gene must be detected in all clusters. The parameter “logfc.threshold” limits testing to genes which show, on average, at least *X*-fold difference (log-scale) between groups of cells. The default test for differential gene expression is “wilcox”. Differentially expressed genes were then displayed on violin plots based on unbiased clustering described above. For TCGA data sets, pancreas cancer cohort was obtained from TCGA (https://tcga-data.nci.nih.gov/tcga/). Clinicopathological characteristics and follow-up survival data were also downloaded from UCSC Xena (https://xena.ucsc.edu/). GSVA is a method that estimates variation of pathway activity over a sample population. GSVA was used to quantify the signature scores of four KPC gene sets (convert mouse to human gene symbols) representing different cell types of each sample in TCGA-PAAD cohort. The ConsensusClusterPlus package was used for consensus clustering and signature subtype screening of GSVA signature scores. GSVA and Consensusclusterplus were performed using the R package GSVA (https://www.bioconductor.org/packages/release/bioc/html/GSVA.html and https://bioconductor.org/packages/release/bioc/html/ConsensusClusterPlus.html).

## Supplementary information

Supplementary figure and legends

Supplementary Table 1

## Data Availability

The data sets of KPC tumor single-cell sequencing that support the findings of this study are available under SRA (Sequence Read Archive) accession number PRJNA634810. All other remaining data that support the findings of this study are available from the authors upon reasonable request.
