# Supplementary material for: Single-cell RNA sequencing reveals that targeting HSP90 suppresses PDAC progression by restraining mitochondrial bioenergetics
Source: Oncogenesis. 2021 Mar 3;10(3):22. doi: 10.1038/s41389-021-00311-4 (PMC7930118; doi:10.1038/s41389-021-00311-4)
Supplement: Supplementary file 1 — Supplementary figure and legends [file 41389_2021_311_MOESM1_ESM.docx]

Supplementary figure and legends

supplementary figure 1


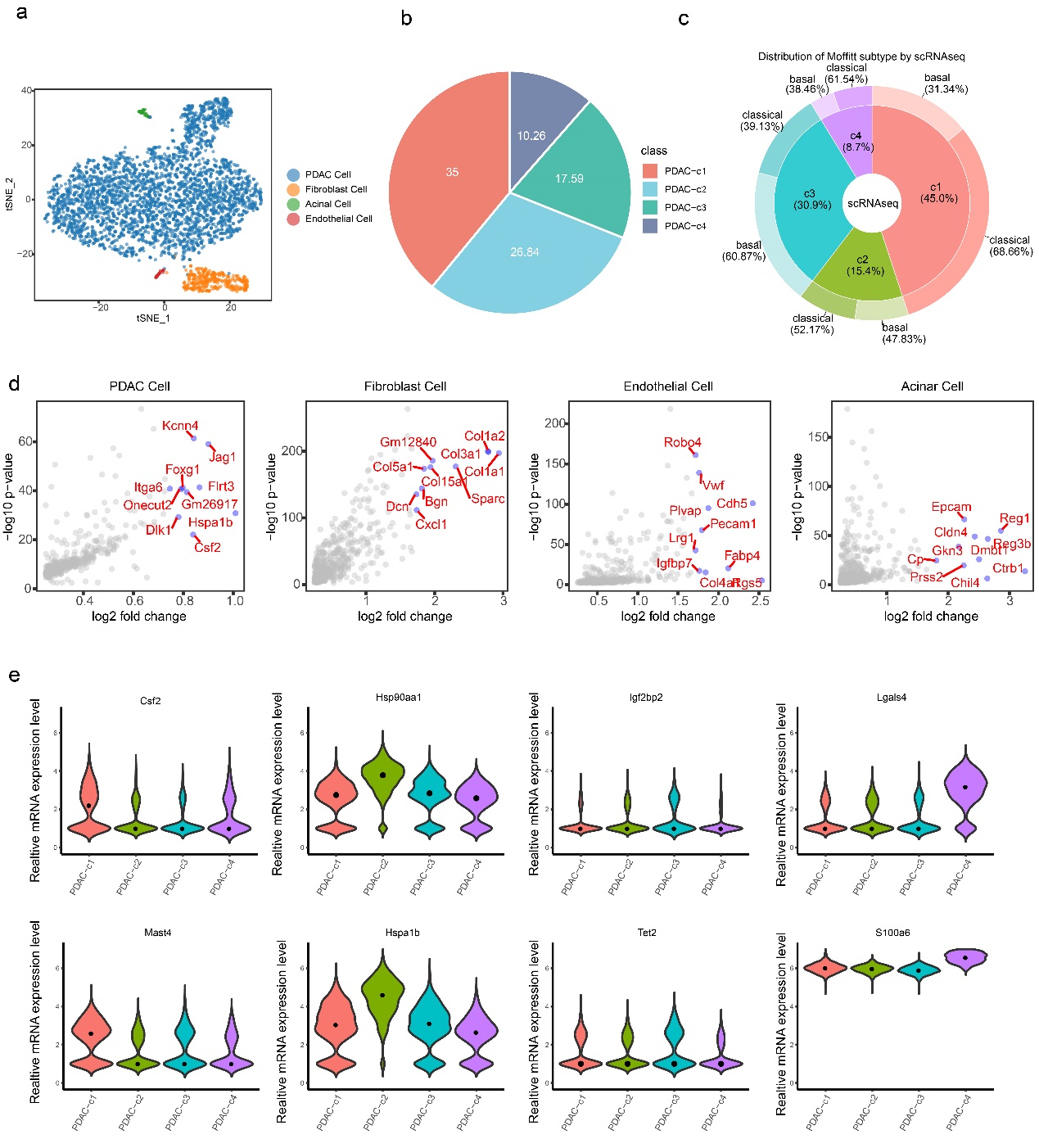


supplymentary figure 1. Single-cell expression profiling and cell typing in pancreatic tumors from KPC a. The t-distributed stochastic neighbor embedding (t-SNE) plot of main cell types in mice PDAC tumor. b. The proportions of each PDAC cancer cell cluster in total PDAC cells. c. The comparison of scRNA clusters and Moffitt clusters. d. Top ten DEG of PDAC cluster, fibroblast cell cluster, endothelial cell cluster and acinar cell cluster. e. Violin plots of PDAC cluster maker genes.

supplementary figure 2


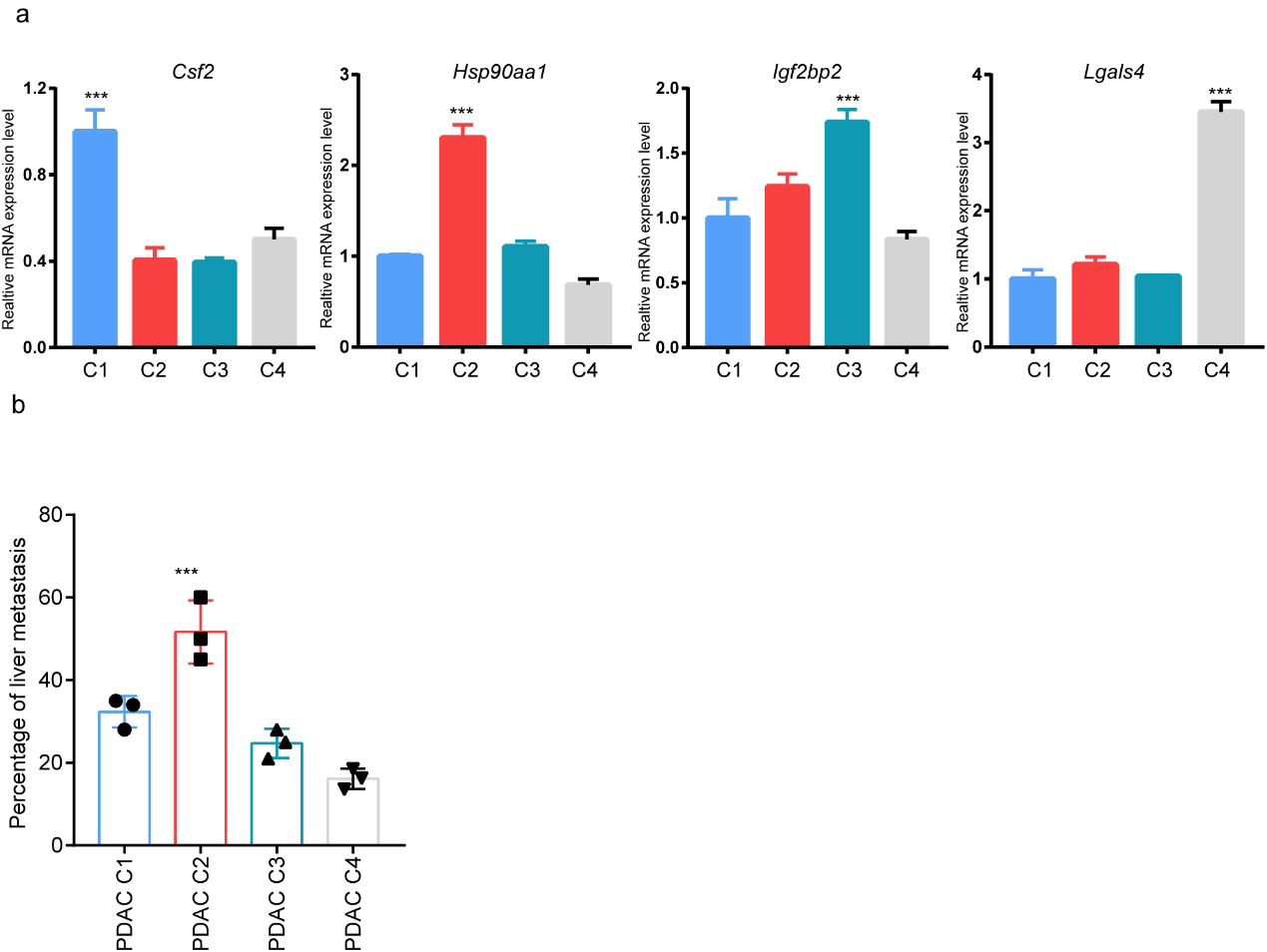


supplementary figure 2. a. The marker genes expression level in the four PDAC cluster cell lines C1, C2, C3, C4. b. The percentage of liver metastasis established by four cluster cells intrasplenical injection derived PDAC liver metastasis model

supplementary figure 3


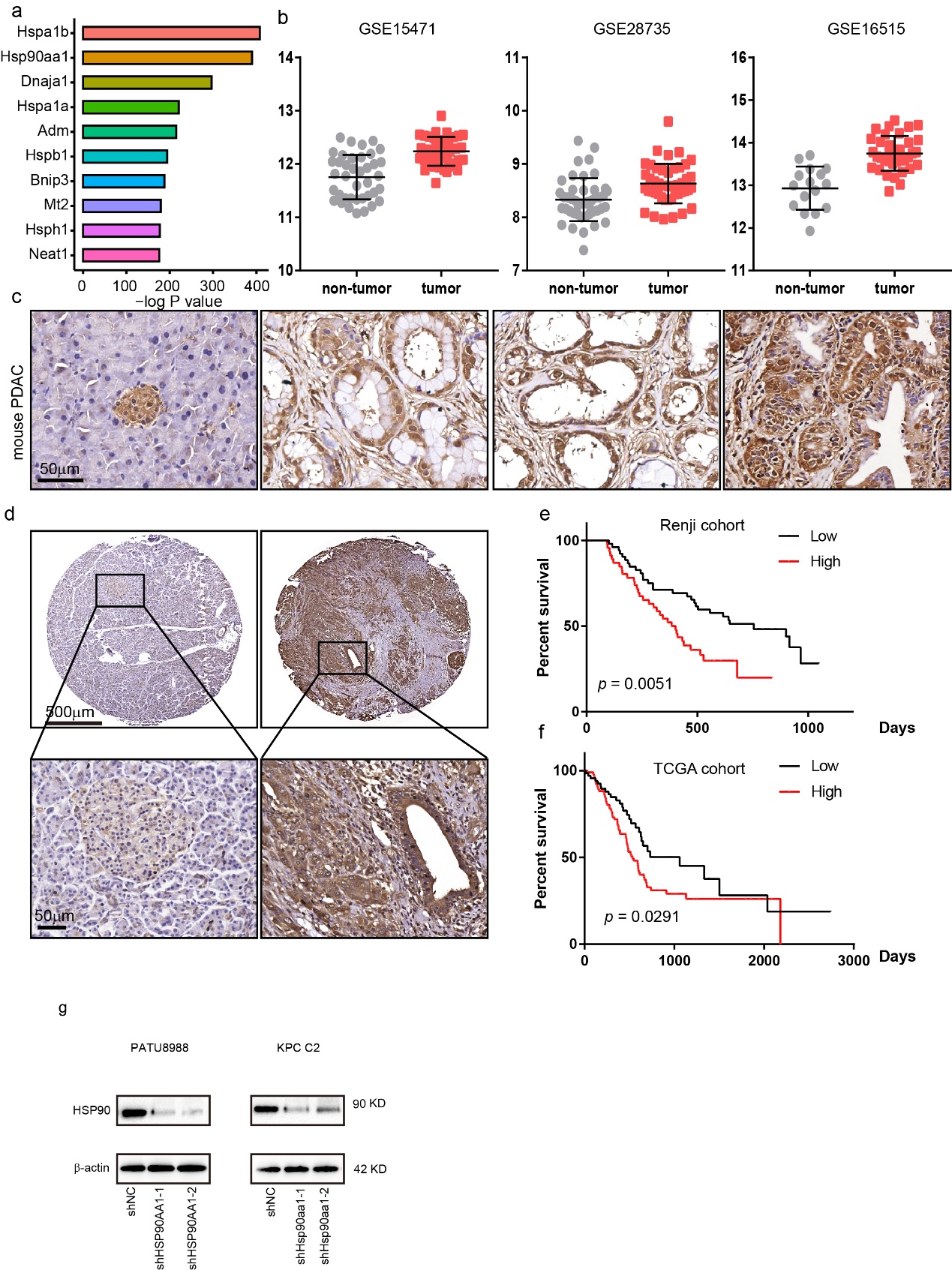


supplementary figure 3. HSP90 is necessary for the tumorgenicity of PDAC cluster 2

a. Top 10 differentially expressed genes of PDAC cluster 2 compared with the other three PDAC clusters. b. The mRNA expression of HSP90AA1 in PDAC tumors and adjacent tissues. c. IHC staining of HSP90 in KPC PDAC tissues in the malignant transformation process. Islet marked with circle. Scale bar, 50 μm. d. Representative IHC staining of HSP90 in a human PDAC tissue array. Islet marked with circle. Scale bar, 500 μm (top), 50 μm (bottom). e-f. Kaplan-Meier survival plot according to low and high HSP90 expression in the Renji cohort(e) and TCGA(f) cohort. g. HSP90 knockdown in PATU8988 and KPC C2

supplementary figure 4


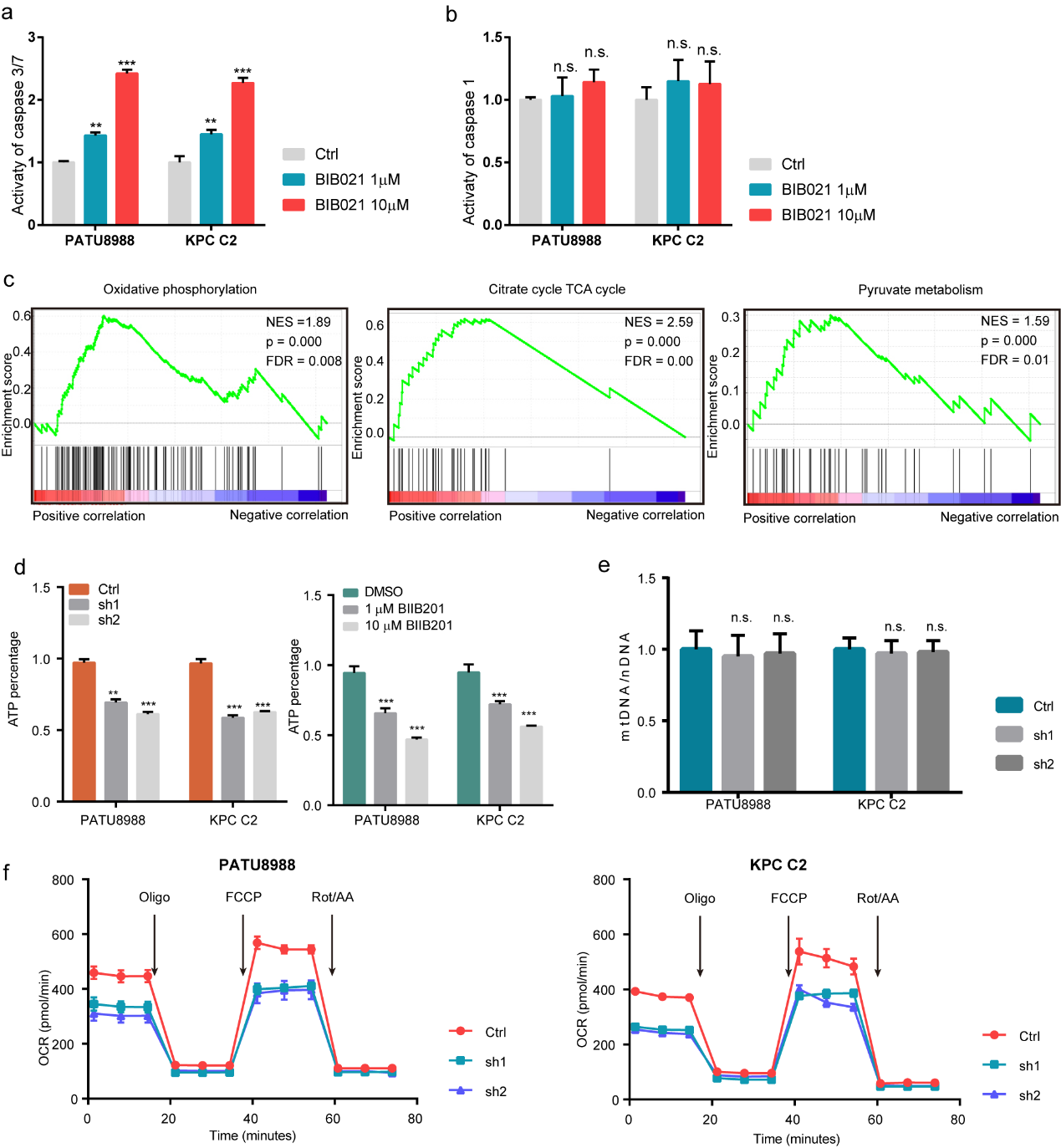


supplementary figure 4. HSP90 inhibition impaired tumor cell mitochondrial bioenergetics.

a. the caspase 3/7 activity of PATU8988 and KPC C2 upon treated with BIIB201. b. The caspase 1 activity of PATU8988 and KPC C2 upon treated with BIIB201. c. The Gene set enrichment analysis plot based on the gene expression profiles of HSP90AA1-high group compared with HSP90AA1-low group. NES, normalized enrichment score. False discovery rate (FDR) was set at 0.25. d. ATP measurement of human and mouse PDAC cells upon HSP90 inhibition. e. mtDNA assay of human and mouse PDAC cells upon HSP90 silencing with shRNA f. OCR measurement in HSP90 knockdown human and mouse PDAC cells.

Supplementary figure 5


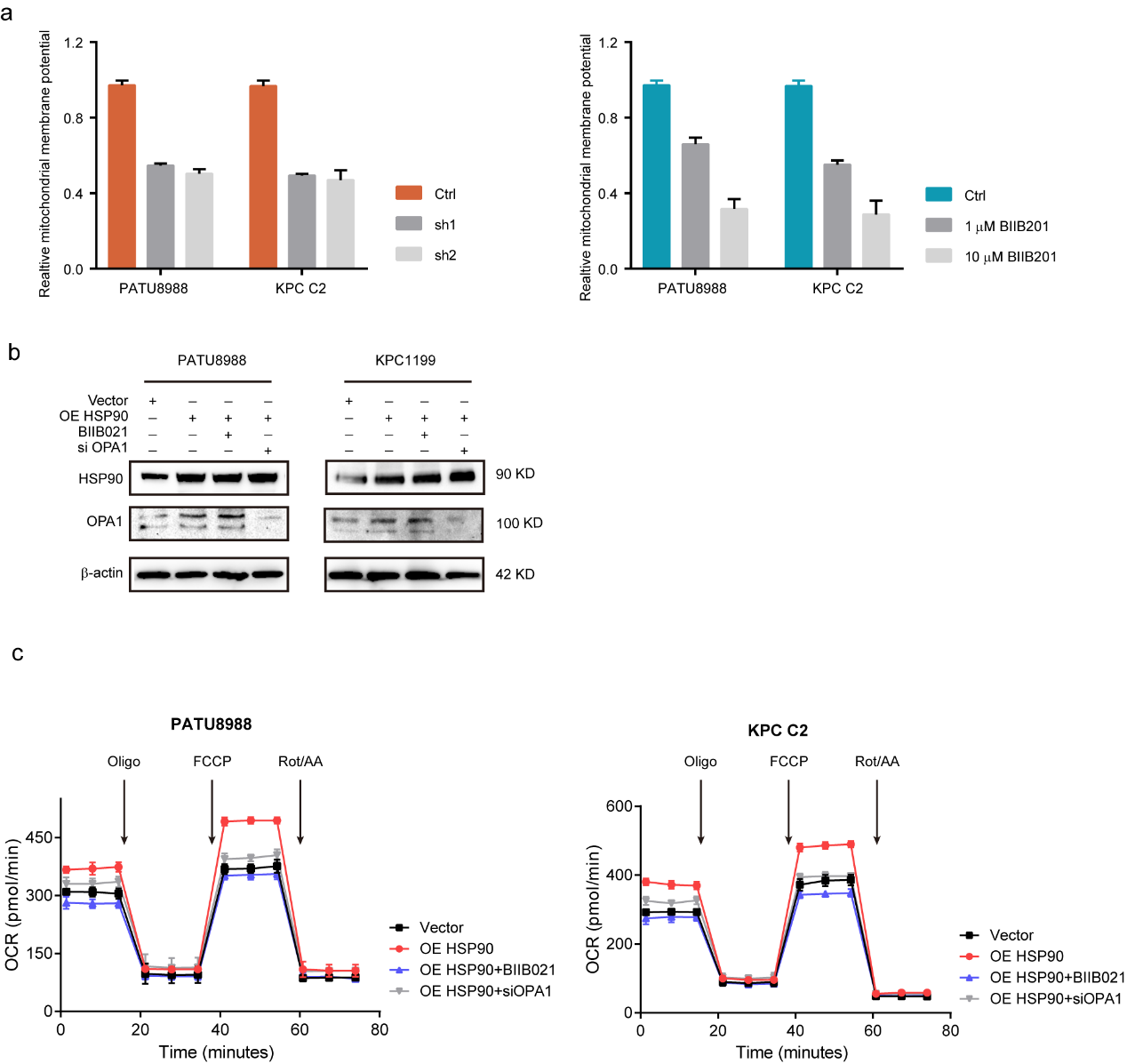


Supplementary figure 5. BIIB021 disrupts the interaction between OPA1 and HSP90.

a. Mitochondrial membrane potential measurement of human and mouse PDAC cells upon HSP90 silencing or blockade with BIIB021. b. western blot analysis of HSP90 and OPA1 expression in PATU8988 and KPC C2 cells overexpressed HSP90 with or without BIIB021 inhibition or siOPA1 treatment. c. OCR measurement in PATU8988 and KPC C2 cells overexpressed HSP90 with or without BIIB021 inhibition or siOPA1 treatment.
